# Mapping Gene Expression in Whole Larval Brains of *Bicyclus anynana* Butterflies

**DOI:** 10.3390/mps8020031

**Published:** 2025-03-13

**Authors:** Tirtha Das Banerjee, Linwan Zhang, Antónia Monteiro

**Affiliations:** Department of Biological Sciences, National University of Singapore, 14 Science Drive 4, Singapore 117543, Singapore; zhang.linwan@u.nus.edu

**Keywords:** multiplex-FISH, HCR3.0, *Bicyclus anynana*, larval brain

## Abstract

Butterfly larvae display intricate cognitive capacities and behaviors, but relatively little is known about how those behaviors alter their brains at the molecular level. Here, we optimized a hybridization chain reaction 3.0 (HCR v3.0) protocol to visualize the expression of multiple RNA molecules in fixed larval brains of the African butterfly *Bicyclus anynana*. We optimized the polyacrylamide gel mounting, fixation, and sample permeabilization steps, and mapped the expression domains of ten genes in whole larval brain tissue at single-cell resolution. The genes included *optomotor blind* (*omb*), *yellow-like*, *zinc finger protein SNAI2-like* (*SNAI2*), *weary* (*wry*), *extradenticle* (*exd*), *Synapsin*, *Distal-less* (*Dll*), *bric-à-brac 1* (*bab1*), *dachshund* (*dac*), and *acetyl coenzyme A acetyltransferase B* (*AcatB*). This method can be used alongside single-cell sequencing to visualize the spatial location of brain cells that change in gene expression or splicing patterns in response to specific behaviors or cognitive experiences.

## 1. Introduction

Insects have relatively small brains but demonstrate remarkable cognitive abilities. Honeybees can remember colors associated with food rewards [1,2,3]. Male moths of the Asian corn borer, *Ostrinia furnacalis*, produce ultrasonic mating calls that exploit the freeze response of female moths to bat calls to improve mating success [4]. Female *Aedes aegypti* mosquitoes utilize multiple sensory cues, such as odor [5,6], thermal [7], carbon dioxide levels [5,6], and taste [8] in combination [7] to locate and feed on human hosts. Yet, an insect brain contains a minuscule fraction of the neurons found in vertebrate brains [9]. With such a large repertoire of sensory modalities and complex behaviors packed into small, compact brains, insects make great model systems for molecular studies of cognition compared to their vertebrate counterparts such as zebrafish and mice.

Butterflies also have a wide variety of senses and cognitive abilities. They respond to visual, auditory, olfactory, vibrational, and gustatory signals and use these signals to search for food, host plants, or suitable mates, to detect predators, and for general navigation [10,11,12,13,14,15,16]. Butterflies can also learn and memorize information. For example, butterflies can learn to prefer specific flower colors [17,18,19] and shapes [20] by training with food rewards, as well as specific host plants or host plant characteristics [20,21,22] after ovipositing on those host plants. Adult females can also learn to prefer novel wing patterns [23] or novel pheromone blends [24] over wild-type patterns or blends if exposed to these cues early in life.

We are starting to understand how novel preferences and behaviors are encoded at the molecular level in the brains of adults or larvae. In a follow-up study to a pheromone blend exposure experiment [24], the brains of the exposed female butterflies showed significant changes in gene expression and splicing patterns a few days later, as analyzed by bulk RNA sequencing on homogenized tissue [25]. Knowledge of where exactly in the brain these changes in gene expression and alternative splicing are taking place is still missing. Such knowledge would allow a more detailed understanding of information processing, memory storage, and behavioral regulation at the molecular level, potentially inspire neural network models [26] and artificial intelligence [27].

To make progress in this field, we optimized a protocol for visualizing gene expression at single-cell resolution in whole-mount larval butterfly brains using hybridization chain reaction 3.0 (HCR v3.0). HCR offers an attractive enzyme-free, isothermal reaction for the localization of mRNA transcripts [28,29]. HCR-based protocol provides key advantages over traditional chromogenic and fluorescent-based in situ methods, such as easy probe design steps, simple methodology, background suppression, multiplexing, and single-cell imaging. Protocols for performing HCR on brain samples of species such as *Drosophila melanogaster* and the jumping ant *Harpegnathos saltator* are in existence [30,31], but no such protocol is available for butterflies. *Bicyclus anynana* larval brains were selected for the development of this protocol, which is adapted from Choi et al. (2018) [32], Toh et al. (2021) [33], and Banerjee et al. (2024) [34]. *B. anynana* butterflies, both at larval and adult stage, have served as a model organism for various behavioral studies [35,36,37] and are amenable to laboratory rearing conditions. The brains of larvae are easier to dissect, which made them the first choice for testing the present experimental protocol. The protocol describes fixation of larval brain samples, immobilization in polyacrylamide gel for 3D multiplexing, and HCR steps.

## 2. Experimental Design

This protocol was developed for *B. anynana* fifth instar larval brains that are approximately 300 µm in diameter for each lobe. Other butterfly brains or other developmental stages may require optimization of incubation timings. All formulations for mixed reagents are described in Section 5.

### 2.1. Materials

#### 2.1.1. Consumables

1. Blade holder (Swann-Morton, Sheffield, England; Cat. No.: 0934)
2. Blades (Swann-Morton, Sheffield, England; Cat. No.: 0115)
3. Dissection silicone plate: Dragon Skin 30 Mold Making Silicone Rubber (Smooth-On, Macungie, PA, USA; Cat. No.: 0751635278417), Petri dish (Sigma-Aldrich, Burlington, MA, USA; Cat. No.: P5981)
4. Filter pipette tips, 10 µL (Biotix, San Diego, CA, USA; Cat. No.: 63300041)
5. Filter pipette tips, 20 µL (Biotix, San Diego, CA, USA; Cat. No.: 63300042)
6. Filter pipette tips, 300 µL (Biotix, San Diego, CA, USA; Cat. No.: 63300045)
7. Filter pipette tips, 1250 µL (Biotix, San Diego, CA, USA; Cat. No.: 63300047)
8. Glass spot plate (Corning, Corning, NY, USA; Cat. No.: 7220-85)
9. Insect pins (BioQuip Products, Rancho Dominguez, CA, USA; Cat. No.: 1208B2)
10. Lint-free tissue, Kimtech Science™ Kimwipes™ Delicate Task Wipes (Kimberly-Clark Professional, Roswell, GA, USA; Cat No.: 34120)
11. Microcentrifuge tubes, 1.5 mL (Eppendorf, Hamburg, Germany; Cat. no.: T9661-500EA)
12. RNaseZap™ RNase Decontamination Solution (ThermoFisher Scientific, Waltham, MA, USA, Cat. No.: AM9780)
13. Straight tweezers, fine (Dumont Switzerland, Montignez, Jura, Switzerland; Cat. No.: 11254-20)
14. Straight tweezers, regular (Dumont Switzerland, Montignez, Jura, Switzerland; Cat. No.: 0203-5-PO)
15. Superfine Vannas scissors, 8 cm (World Precision Instruments, Sarasota, FL, USA; Cat. No.: 501778)

#### 2.1.2. Reagents

1. Acrylamide/bis-acrylamide, 19:1 (Sigma-Aldrich, Burlington, MA, USA; Cat. No.: A2917)
2. Ammonium persulfate (APS) (Sigma-Aldrich, Burlington, MA, USA; Cat. No.: A7460)
3. Calcium chloride (CaCl_2_) (Sigma-Aldrich, Burlington, MA, USA; Cat. No.: C4901)
4. Citric acid (Sigma-Aldrich, Burlington, MA, USA; Cat. No.: 251275)
5. DAPI (Sigma-Aldrich, Burlington, MA, USA; Cat. No.: D9542)
6. Denhardt’s solution, 50× (ThermoFisher, Massachusetts, USA; Cat. No.: 750018)
7. Dextran sulfate (Sigma-Aldrich, Burlington, MA, USA; Cat. No.: D6001)
8. Diethyl pyrocarbonate (DEPC) (Sigma-Aldrich, Burlington, MA, USA; Cat. No.: D5758)
9. Dimethyl sulfoxide (DMSO) (Sigma-Aldrich, Burlington, MA, USA; Cat. No.: D8418)
10. DNase I, 1 U/µL (ThermoFisher Scientific, Waltham, MA, USA, Cat. No.: EN0521)
11. Ethylenediaminetetraacetic acid (EDTA) (Sigma-Aldrich, Burlington, MA, USA; Cat. No.: E9884)
12. Formaldehyde, 37% (Sigma-Aldrich, Burlington, MA, USA; Cat. No.: F8775)
13. Formamide (Sigma-Aldrich, Burlington, MA, USA; Cat. No.: F7503)
14. Glycerol (Sigma-Aldrich, Burlington, MA, USA; Cat. No.: G7893)
15. H1, H2 hairpins (Molecular Instruments, Los Angeles, CA, USA)
16. Heparin (Sigma-Aldrich, Burlington, MA, USA; Cat. No.: H3393)
17. Magnesium chloride (MgCl_2_) (Sigma-Aldrich, Burlington, MA, USA; Cat. No.: M8266)
18. N,N,N′,N′-Tetramethylethylenediamine (TEMED) (Sigma-Aldrich, Burlington, MA, USA; Cat. No.: T9281)
19. Primary oligos, 100 µM in IDTE, pH 8.0 (Molecular Instruments, CA, USA; Integrated DNA Technologies, Coralville, IA, USA)
20. Potassium phosphate dibasic (K_2_HPO_4_) (Sigma-Aldrich, Burlington, MA, USA; Cat. No.: P3786)
21. Potassium phosphate monobasic (KH_2_PO_4_) (Sigma-Aldrich, Burlington, MA, USA; Cat. No.: P0662)
22. Sodium chloride (NaCl) (Sigma-Aldrich, Burlington, MA, USA; Cat. No.: S9888)
23. Sodium dodecyl sulfate (SDS) (Sigma-Aldrich, Burlington, MA, USA; Cat. No.: 436143)
24. Sodium hydroxide (NaOH) pellets (Sigma-Aldrich, Burlington, MA, USA; Cat. No.: 221465)
25. Tris-hydrochloride (Tris-HCl), pH 7.5 (Sigma-Aldrich, Burlington, MA, USA; Cat. No.: 10812846001)
26. Trisodium citrate (Sigma-Aldrich, Burlington, MA, USA; Cat. No.: S1804)
27. TWEEN 20 (Sigma-Aldrich, Burlington, MA, USA; Cat. No.: P1379)

### 2.2. Equipment

1. Autoclave (HIRAYAMA, Saitama, Japan; Product ID: HV-110)
2. Confocal microscope, Olympus FLUOVIEW FV3000 confocal LSM (Olympus Life Science, Waltham, MA, USA; Product ID: FV3000)
3. Incubating rocking shaker (OHAUS, Parsippany, NJ, USA; Product ID: ISRK04HDG)
4. Micropipette, 0.1–2.5 µL (Eppendorf, Hamburg, Germany; Cat. no.: 3123000012)
5. Micropipette, 0.5–10 µL (Eppendorf, Hamburg, Germany; Cat. no.: 3123000020)
6. Micropipette, 20–200 µL (Eppendorf, Hamburg, Germany; Cat. no.: 3123000055)
7. Micropipette, 100–1000 µL (Eppendorf, Hamburg, Germany; Cat. no.: 3123000063)
8. Milli-Q^®^ Ultrapure Water Systems (Merck Millipore, Burlington, MA, USA)
9. Stereo microscope, ZEISS Stemi 305 (ZEISS, Oberkochen, Germany; Product ID: Stemi 305)

## 3. Procedure

### 3.1. Probe Design

Probes against the 10 genes described in the present protocol were designed using the Excel sheets described in Banerjee et al., 2024 [34]. Briefly, raw CDS files of the genes were downloaded from NCBI. The sequences were added to the CDS_gene_extractor file, which selects 25 bp split probe binding sites and reverse complements them. Six to eight pairs of the sequences were selected and added to the amplifier_adder file, which adds the specific secondary binding sequences (B1, B2, or B3) to create the final probes.

### 3.2. Dissection and Preparation of Larval Brains for HCR

#### 3.2.1. Dissection and Fixation (~3 h for 1 Sample, +20 Min for Every Additional Sample)

1. Dissect fifth instar larval brains in 1× phosphate-buffered saline (PBS) at room temperature (RT). For detailed information on the dissection protocol for larval brains, refer to the published protocol by Toh et al. (2021), Section 5 [33].
2. In a 1.5 mL microcentrifuge tube, prepare 500 µL of fresh 4% phosphate-buffered formaldehyde (PFA). Prepare one tube for every three larval brains processed.
3. Using a P1000 micropipette, gently aspirate the dissected brains and transfer them into the microcentrifuge tubes containing 4% PFA. Each tube should contain no more than three brains.

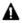
 **CRITICAL STEP** Samples should be aspirated gently to prevent damage. One technique is to use the volume adjustment ring on the pipette instead of the plunger to aspirate samples gently. This is accomplished by first adjusting the set volume on the pipette to the lowest setting (100 µL), immersing the pipette tip into the solution next to the samples, and then smoothly rotating the volume adjustment ring toward a higher volume setting. The tip of the pipette can also be cut using a pair of scissors for a larger inlet diameter.

4. Incubate the samples on a shaker for 1.5 h at RT with gentle agitation (~60 rpm in a horizontal shaker). For smaller tissues, fixation time can be reduced.
5. Wash the samples three times with 500 µL of 1× phosphate-buffered saline with TWEEN 20 (1× PBST) for 10 min each, at RT.

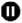
 **PAUSE STEP** The samples can be stored in 1× PBST at 4 °C for up to 3 days.

#### 3.2.2. Permeabilization and Post-Fixation (~2.5 h)

6. Replace 1× PBST with 500 µL of detergent solution.
7. Incubate the samples on a shaker for 30 min at RT with gentle agitation.
8. Rinse the samples two times with 500 μL of 1× PBST each, at RT.
9. Transfer the samples into microcentrifuge tubes containing 500 μL of fresh 4% PFA.
10. Incubate the samples on a shaker for 20 min at RT with gentle agitation.
11. Wash the samples three times with 500 µL of 1× PBST for 10 min each, at RT.
12. Wash the samples two times with 500 µL of 5× saline–sodium citrate buffer with TWEEN 20 (SSCT) for 3 min each, at RT.
13. Transfer the samples into microcentrifuge tubes containing 500 μL of 30% probe hybridization buffer.
14. Incubate the samples on an incubating shaker for 30 min at 37 °C with gentle agitation.

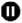
 **PAUSE STEP** The samples can be stored in 30% probe hybridization buffer at 4 °C for up to 3 weeks.

### 3.3. Embedment of Fixed Brains in Polyacrylamide Gel

#### 3.3.1. Preparation for Gel Embedment (~30 min)

1. In a 1.5 mL microcentrifuge tube, add 200 µL of 40% acrylamide solution, 400 µL of 2.0 M NaCl, 60 µL of 1.0 M Tris-HCl, pH 7.5, 325 µL of DEPC H_2_O, and 4 µL of TEMED for 1 mL of gel solution.

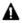
 **CRITICAL STEP** The gel solution should be prepared fresh. A total of 1 mL of solution is sufficient for five gel castings. Invert or pipette gently to mix. Do not vortex the gel solution or any of the components.

**Figure 1:**
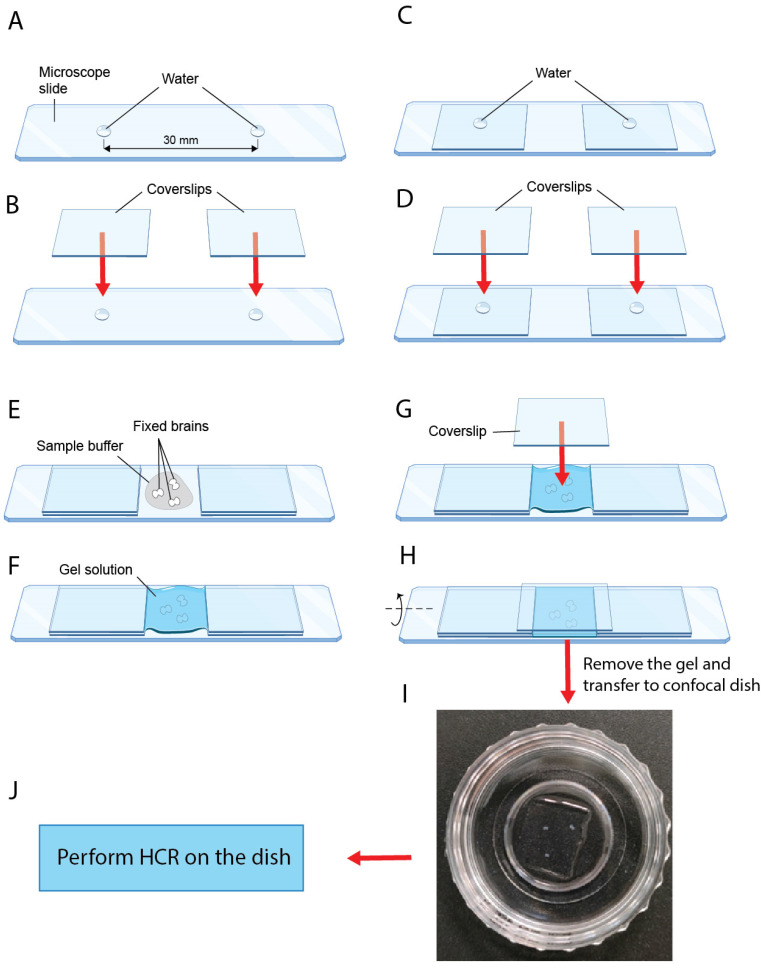
**Illustrated depiction of preparation steps for gel embedment of larval brains.** (**A**) Two drops of 0.5 μL of DEPC H_2_O are placed approximately 30 mm apart on the microscope slide. (**B**) Coverslips are lowered onto the water drops. (**C**) Another pair of water drops are placed on top of the coverslips. (**D**) Another pair of coverslips are lowered onto the water drops. (**E**) Brain samples are placed between the coverslip stacks. (**F**) Sample buffer is replaced by gel solution. (**G**) A coverslip is lowered on top of the gel solution. (**H**) The microscope slide may be rotated longitudinally to remove trapped bubbles. (**I**) Gel-embedded brain samples in a confocal dish. (**J**) HCR is performed on the confocal dish.

2. Allow the gel solution and 10% APS stock solution to normalize to RT.
3. If more than one gel piece is to be cast using the stock gel solution, for every gel piece, prepare a 1.5 mL microcentrifuge tube containing 2 µL of 10% APS. Otherwise, skip this step.
4. Clean a microscope slide, five coverslips, tweezers, and scalpel blade with RNaseZap, using KimWipes.
5. Using a P2 micropipette, dispense 0.5 μL of DEPC H_2_O onto the microscope slide, approximately 30 mm apart (Figure 1A).
6. Place a coverslip on top of each droplet of water, with a clearance of about 12–16 mm between the coverslips (Figure 1B). Press the coverslips firmly down onto the droplets of water to temporarily adhere the coverslips to the microscope slide.
7. Dispense 0.5 μL of DEPC H_2_O using a P2 micropipette onto the center of each of the coverslips (Figure 1C).
8. Stack another coverslip onto each of the coverslips on the slide, pressing firmly down to adhere the coverslips to each other (Figure 1D).

#### 3.3.2. Gel Embedment of Larval Brains (~30 min)

9. Using a P1000 micropipette, transfer the desired number of fixed brain samples onto the microscope slide in between the coverslips (Figure 1E).
10. Using a P200 micropipette, remove any excess buffer solution on the microscope slide.
11. If only one gel piece is to be cast from the stock gel solution, add 10 µL of 10% APS to the gel solution prepared in Step 1. If more than one gel pieces are needed, add 200 µL of the gel solution in Step 1 to a microcentrifuge tube containing 2 µL of 10% APS (prepared in Step 3). Minor fluctuations in volume do not affect performance.

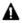
 **CRITICAL STEP** After APS is added to the gel solution, the gel polymerizes in the microcentrifuge tube within 1 min. Make sure to only proceed with this step after all other preparation steps are complete. Work quickly from this step onward until Step 18 to prevent the gel solution from polymerizing prematurely.

12. Invert or pipette three times to mix.
13. Immediately transfer up to 200 μL of gel solution onto the brain samples. The area between the coverslips should be mostly filled with gel solution (Figure 1F).
14. Under a stereomicroscope, separate and rearrange the brain samples to the desired configuration using precision tweezers.
15. Lower the last cleaned coverslip onto the samples so that the ends of the coverslip are supported by the stacked coverslips (Figure 1G).
16. Gently wipe away any excess gel solution using a lint-free tissue.
17. Gently tilt the microscope slide along the longitudinal axis to remove any trapped bubbles (Figure 1H).
18. Allow the gel to polymerize fully for 15 min.
19. Using a pair of tweezers, gently remove the coverslips surrounding the gel layer.
20. Cut the gel to the desired size using a scalpel blade. The gel should fit comfortably in the imaging area of the confocal dish.
21. Using a P200 micropipette, dislodge the gel from the microscope slide by flushing it with 5× SSCT. This can be accomplished by gently inserting the pipette tip between the edge of the gel and the microscope slide, then dispensing 5× SSCT into the gap between the gel layer and the microscope slide.
22. Transfer the gel layer to a confocal dish using a pair of tweezers (Figure 1I).
23. Rinse the gel two times with 500 μL of 5× SSCT each, at RT.
24. To cast more gel pieces, repeat Steps 4–23. Otherwise, proceed to HCR v3.0.

### 3.4. HCR v3.0 Signal Capture

1. Incubate the samples in 500 μL of the desired primary oligo mixture for 20–24 h in RT, followed by 1 h in an incubating shaker at 37 °C with gentle agitation.
2. Preheat 30% probe wash buffer to 37 °C.
3. Wash the samples eight times with 500 μL of 30% probe wash buffer for 15 min each at 37 °C.
4. Wash the samples two times with 500 μL of 5× SSCT for 3 min each at RT.
5. Incubate the samples on a shaker in 500 µL of amplification buffer at RT with gentle agitation.
6. Replace the amplification buffer with 200 µL of the corresponding secondary oligo mixture.

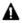
 **CRITICAL STEP** From this step onward, until after confocal imaging, all steps should be performed in the dark as much as possible.

7. Incubate the samples for 20–24 h in RT, followed by 2 h in an incubating shaker at 37 °C with gentle agitation.
8. Wash the samples six times with 500 μL of 5× SSCT for 10 min each at 37 °C.
9. **OPTIONAL STEP** If DAPI staining is desired, add 500 μL of DAPI buffer and incubate for 20 min at RT with gentle agitation. If not, skip Steps 9 and 10.
10. **OPTIONAL STEP** Wash the samples two times with 500 μL of 5× SSCT for 3 min each, at RT.
11. Remove any excess buffer solution using a P200 pipette.
12. Add a small amount of 60% glycerol, at a sufficient volume to fully coat the gel surface but not enough to cause the gel to shift around in the confocal dish.
13. Wait for 15 min in RT for the samples to stabilize before imaging.

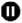
 **PAUSE STEP** The samples can be stored in 1 mL of 60% glycerol at 4 °C for more than 2 weeks if not imaged immediately. Before imaging, allow the samples to come to RT for at least 30 min and remove any excess 60% glycerol to prevent the samples from shifting positions during imaging.

14. For imaging, the confocal dish with brains embedded in the polyacrylamide gel was mounted on an Olympus fv3000 microscope (Tokyo, Japan). Z-section (~5 µm) scans of the entire brain were carried out at 2k or 4k resolution using four channels: DAPI, AF488, AF546, and AF647. Images were later processed and stitched via Olympus floview or Imaris viewer.

### 3.5. Signal Removal (from Banerjee et al., 2024 [34])

This section is **optional** and should only be performed if more gene targets are to be multiplexed in one sample than what can be imaged in one round of HCR.

1. Rinse the samples two times with 500 μL of 5× SSCT at RT.
2. Wash the samples three times with 500 μL of signal wash buffer 1 for 3 min each at RT.
3. Incubate the samples on an incubating shaker in 500 μL of signal removal solution for 50 min at 37 °C with gentle agitation.
4. Wash the samples two times with 500 μL of signal wash buffer 2 for 15 min each at 37 °C.
5. Wash the samples four times with 500 μL of signal wash buffer 1 for 3 min each at 37 °C.
6. **OPTIONAL STEP** Image the samples to ensure all previous signals (except for DAPI staining, if any) have been removed. If signals are still present, repeat from Step 1, adjusting the incubation time in Step 3 as needed.
7. Wash the samples two times with 500 µL of 5× SSCT for 3 min each, at RT.
8. Incubate the samples on an incubating shaker in 500 μL of 30% probe hybridization buffer for 30 min at 37 °C with gentle agitation.

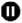
 **PAUSE STEP** The samples can be stored in 30% probe hybridization buffer at 4 °C for up to 3 weeks.

9. For the next round of HCR, repeat from Section 3.2, Step 1.

## 4. Expected Results

The successful embedding of brain samples should produce a gel layer, as shown in Figure 1I. Common problems and possible solutions are listed in Table 1.

With this protocol, we were able to obtain high-quality 3D confocal scans of *B. anynana* larval brains for various gene targets. Example confocal images obtained are shown below in Figure 2, Figure 3, Figure 4 and Figures S1–S5.

Video S1. Three-dimensional expression of *omb* (red), *Synapsin* (green), *Dll* (gray), and DAPI (blue) in the larval brain (anterior domain) of *B. anynana*.

Video S2. Three-dimensional expression of *omb* (red), *Synapsin* (green), *Dll* (gray), and DAPI (blue) in larval brain (posterior domain) of *B. anynana*.

Video S3. Three-dimensional expression of *omb* in the larval brain (left lobe) of *B. anynana* (DAPI in blue).

Video S4. Three-dimensional expression of *Synapsin* in the larval brain (left lobe) of *B. anynana* (DAPI in blue).

Video S5. Three-dimensional expression of *Dll* in the larval brain (left lobe) of *B. anynana* (DAPI in blue).

Video S6. Three-dimensional expression of *SNAI2* in the larval brain (left lobe) of *B. anynana* (DAPI in blue).

Video S7. Three-dimensional expression of *exd* in the larval brain (left lobe) of *B. anynana* (DAPI in blue).

Video S8. Three-dimensional expression of *AcatB* in the larval brain (left lobe) of *B. anynana* (DAPI in blue).

Video S9. Three-dimensional expression of *dac* in the larval brain (left lobe) of *B. anynana* (DAPI in blue).

Video S10. Three-dimensional expression of *yellow-like* in the larval brain (left lobe) of *B. anynana* (DAPI in blue).

Video S11. Three-dimensional expression of *wry* in the larval brain (left lobe) of *B. anynana* (DAPI in blue).

Video S12. Three-dimensional expression of *bab1* in the larval brain (left lobe) of *B. anynana* (DAPI in blue).

Note: High-resolution tif files are provided in the Supplementary Materials and can be opened using Imaris Viewer (Oxford Instruments).

The following genes were tested in *B. anynana* larval brains. Additional details on gene sequences can be found in the Supplementary Excel File.

- *optomotor-blind* (*omb*)

**Figure 2:**
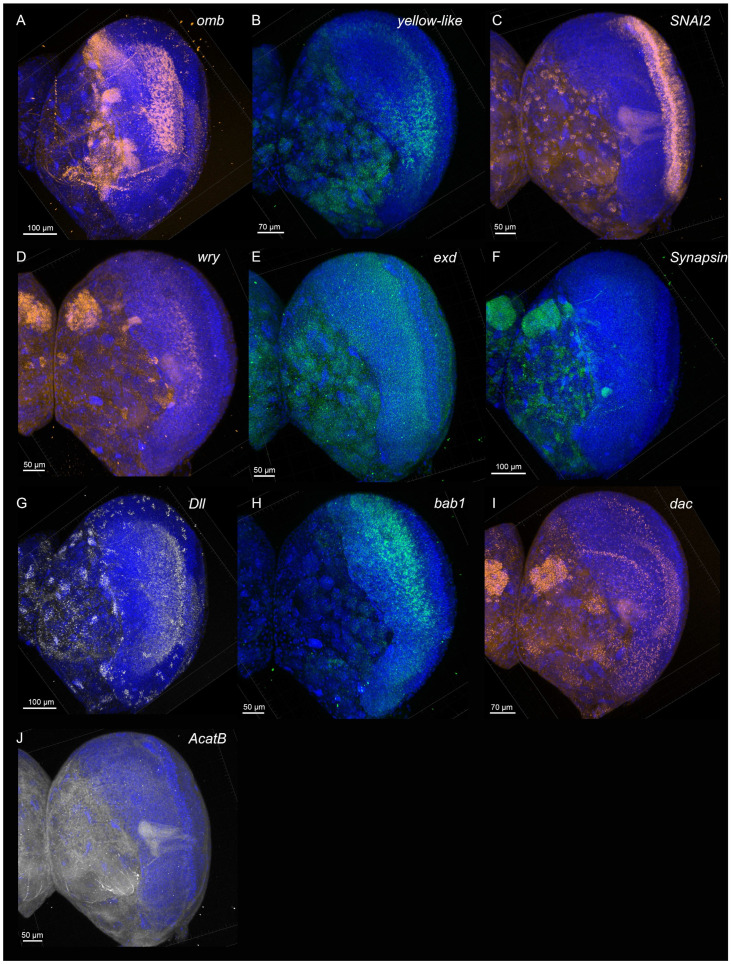
Expression (merged z-stack) of (**A**) *optomotor blind* (*omb*), (**B**) *yellow-like*, (**C**) *Zinc finger protein SNAI2-like* (*SNAI2*), (**D**) *weary* (*wry*), (**E**) *extradenticle* (*exd*), (**F**) *Synapsin*, (**G**) *Distal-less* (*Dll*), (**H**) *bric-à-brac 1* (*bab1*), (**I**) *dachshund* (*dac*), and (**J**) *acetyl coenzyme A acetyltransferase B* (*AcatB*) across 150–200 µm sections (5 µm slices) of fifth instar larval brains of *Bicyclus anynana*.

**Figure 5:**
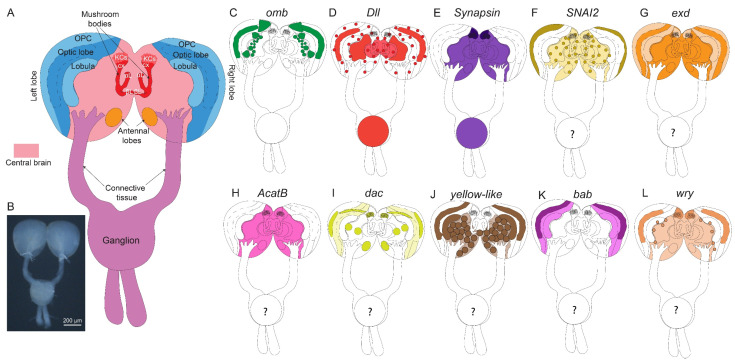
**Illustration of a *B. anynana* larval brain and domains of gene expression (anterior view).** (**A**) A simplified illustration of a larval brain using nomenclature from Egger et al. (2011) [60], Ito et al. (2014) [61], Sehadová et al. (2023) [62], Wang et al. (2024) [63]. (**B**) **A darkfield image of a dissected larval brain**. Expression of (**C**) *omb*, (**D**) *Dll*, (**E**) *Synapsin*, (**F**) *SNAI2*, (**G**) *exd*, (**H**) *AcatB*, (**I**) *dac*, (**J**) *yellow-like*, (**K**) *bab1*, and (**L**) *wry*. KCs: Kenyon Cells; cx: calyx; αL: alpha-Lobe; βL: beta-Lobe; OPC: Outer Proliferating Center.

In *Drosophila*, *omb is* involved in the development of the optic lobes [38]. Knockout mutations in *omb* cause severe depletion of neuropil volume, sometimes even causing a complete loss of neuropil of the optic lobes. In *B. anynana*, *omb* expression was observed in the optic lobes and in the lobula of the larval brains. Stronger *omb* expression was also observed in the distal domains of the central brain (Figure 2A, Figure 4B,E, Figure 5C, Figure S2, S3A,E,F,J, S4 and S8A–E; Videos S1–S3).

- *Distal-less* (*Dll*)

In *Drosophila*, *Dll* has been shown to be expressed in the central nervous system and peripheral nervous system and has been proposed to play a role in olfaction [39]. In adult *Drosophila* brain, expression was observed in the optic lobes, central brain, and antennal lobes [39]. In *B. anynana*, *Dll* expression was observed in the optic lobes and in the ventromedial and ventrolateral neuropils of the central brains. *Dll* was also expressed in the ganglion. Small clusters of *Dll* were observed throughout the brain (Figure 2G, Figure 4C,F, Figure 5D, Figure S2, S3B,E,G,J and S8A″–E″; Videos S1, S2 and S5).

- *Synapsin*

*Synapsin* is a useful gene for the visualization of brain structure and organization because it is highly conserved across a large variety of species and has relatively high expression levels in the nervous system. Synapsin proteins are present in all organisms with a nervous system [40], and they regulate neurotransmitter release by maintaining the pool of synaptic vesicles [41]. *Synapsin* null mutants exhibit reduced capability to maintain synaptic response to stimulation in *Drosophila* [42], revealing a role in synaptic vesicle reuptake and reserve pool replenishment. In *B. anynana*, *Synapsin* expression was strongly observed throughout the central brain and in the ganglion (Figure 2F, Figure 4A,D, Figure 5E, Figure S2, S3C,E,H,J, S4 and S8A’–E’; Videos S1, S2 and S4).

- *Zinc finger protein SNAI2-like* (*SNAI2*)

**Figure 3:**
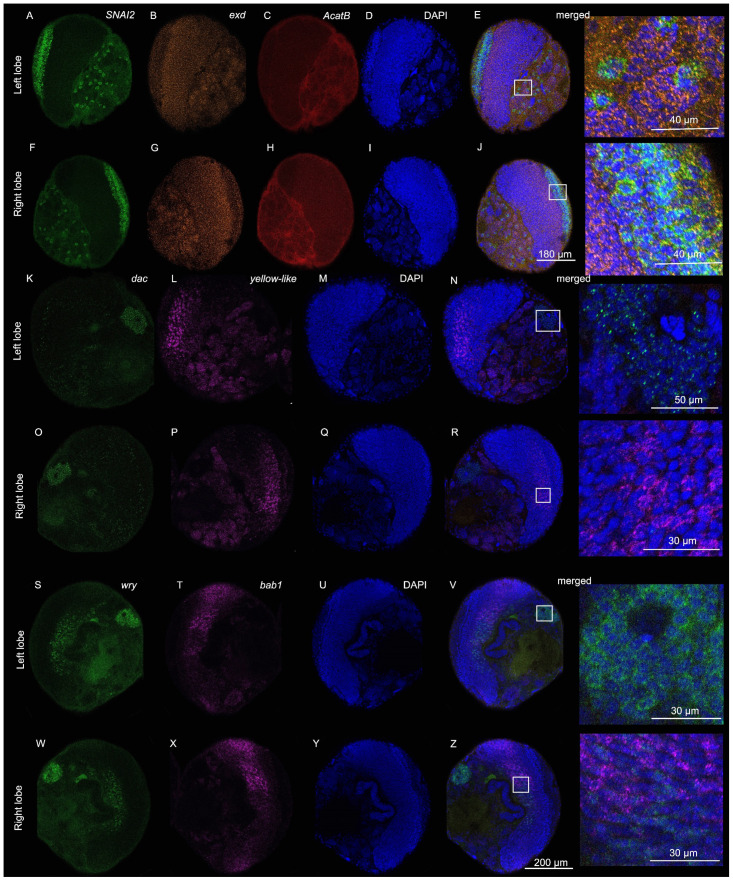
**Expression of *Zinc finger protein SNAI2-like* (*SNAI2*)**, ***extradenticle* (*exd*)**, ***acetyl-CoA acetyltransferase B* (*AcatB*)**, ***dachshund* (*dac*)**, ***yellow-like****, **wry***, **and *bric-à-brac 1* (*bab1*) in single larval brain lobes (~5 µm section) of *B. anynana*.** (**A**–**J**) First brain sample displays signals for (**A**,**F**) *SNAI2*, (**B**,**G**) *exd*, (**C**,**H**) *AcatB*, (**D**,**I**) DAPI, and (**E**,**J**) merged channels, with highlighted sections zoomed in. (**K**–**R**) Second brain sample displays signals for (**K**,**O**) *dac*, (**L**,**P**) *yellow-like*, (**M**,**Q**) DAPI, and (**N**,**R**) merged channels, with highlighted sections zoomed in. (**S**–**Z**) Third brain sample displays signals for (**S**,**W**) *wry*, (**T**,**X**) *bab1*, (**U**,**Y**) DAPI, and (**V**,**Z**) merged channels, with highlighted sections zoomed in.

**Figure 4:**
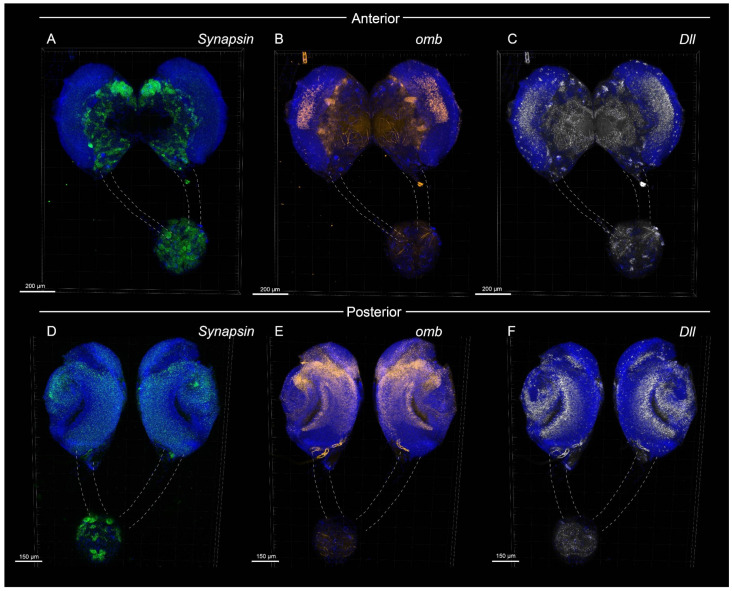
**Expression of *Synapsin*, *optomotor blind* (*omb*)**, **and *Distal-less* (*Dll*) in a larval brain of *B. anynana.*** Dotted lines indicate connective tissues. (**A**–**C**) Anterior domain of the larval brain, showing signals for (**A**) *Synapsin*, (**B**) *omb*, and (**C**) *Dll*, (**D**–**F**) Posterior domain of the larval brain, showing signals for (**D**) *Synapsin*, (**E**) *omb*, and (**F**) *Dll*.

*SNAI2* is a member of the Snail family of zinc-finger transcription factors [43]. In *Drosophila*, Snail genes have been implicated in positively influencing transcriptional activity [44]. *SNAI2* expression was observed in cell clusters throughout the central brain and stronger expression along the distal domain of the optic lobes of the larval brains (Figure 2C, Figure 3A,E,F,J, Figure 5F and Figure S5; Video S6).

- *Extradenticle* (*exd*)

Extradenticle proteins are regulators of homeotic gene activity [45]. In particular, along with other homeotic selector proteins, Extradenticle establishes segmental identities in *Drosophila* [46]. It serves as a cofactor for HOX proteins for regulation of target specificity [47]. *exd* expression was observed throughout the two lobes of the brain with slightly lower expression along cells at the distal and proximal edges of the optic lobes of the larval brains (Figure 2E, Figure 3B,E,G,J, Figure 5G and Figure S5; Video S7).

- *Acetyl-CoA acetyltransferase B* (*AcatB*)

Acetyl-CoA acetyltransferase catalyzes the formation of acetoacetyl-CoA from acetyl-CoA [48]. In insects, acetyltransferases are responsible for the inactivation of neurotransmitters [49], as well as other functions such as melanin synthesis [50]. In *B. anynana*, *AcatB* expression was observed throughout the central larval brain (Figure 2J, Figure 3C,E,H,J, Figure 5H and Figure S5; Video S8).

- *dachshund* (*dac*)

Dachshund is a highly conserved nuclear protein related to the Ski/Sko family of corepressors [51]. In *Drosophila*, *dac* plays key roles in eye and brain development [51], where *dac* plays an important role in retinal and mushroom body development [51,52]. In the *Drosophila* brain, Dac is strongly expressed in the Kenyon cells of the mushroom bodies [52]. In *B. anynana*, *dac* is associated with eye developmental plasticity [53]. *dac* expression was observed in the Kenyon cells of the mushroom bodies, in groups of cells in the central brain, antennal lobes, and optics lobes of the larval brains (Figure 2I, Figure 3K,N,O,R, Figure 5I and Figure S6; Video S9).

- *yellow-like*

The functions of *yellow* genes are still largely unknown. *Yellow* genes are likely to be involved in melanin synthesis [54,55] and in the regulation of behavior [56]. Mutations in *B. anynana yellow* produced individuals that displayed changes in coloration and in courtship behavior [56]. *yellow-like* expression was observed in the central brain (except in the ventromedial neuropils and superior neuropils), and stronger expression was observed in a group of cells in the optics lobes of the larval brains (Figure 2B, Figure 3L,N,P,R, Figure 5J and Figure S6; Video S10).

- *bric à brac 1* (*bab1*)

In *Drosophila*, *bab1* has been shown to play an important function in defining the mushroom body size and shape [57]. In *B. anynana*, the *bab1* expression was observed in bands in the optics lobes of the larval brains (Figure 2H, Figure 3T,V,X,Z, Figure 5K and Figure S7; Video S12).

- *weary* (*wry*)

The Notch signaling pathway plays vital roles at various stages in *Drosophila* embryonic neurogenesis [58]. Weary (Wry) is a Notch ligand that has been shown to play an important role in the normal heart function of adult *Drosophila* [59]. In *B. anynana*, *wry* expression was observed in the anterior domains of the central brain and in the optics lobes of the larval brains. Small clusters of expression were observed throughout the central brain. (Figure 2D, Figure 3S,V,W,Z, Figure 5L and Figure S7; Video S11).

Note: Even though HCR3.0 allows specific deep tissue binding of the probes, several key points were considered during the experiment, interpretation, and analysis of the spatial expression patterns. HCR3.0 is highly specific due to the split probe design and provides exceptional background suppression. However, during our experiments, we have sometimes observed nonspecific fluorescent clusters, which are either due to autofluorescence or likely due to the accumulation of secondary probes, which were not washed away due to the uneven nature of the tissue. Such images and data were excluded. Furthermore, since the brain is a 3D tissue, certain washes might produce an uneven effect of the fluorescent signal. We have increased the number of primary and secondary wash steps, as well as increased the washing temperature to 37 °C to address this issue.

## 5. Reagents Setup

All reagents and buffers used in the protocol are described in Table 2.

## Figures and Tables

**Table 1:** Troubleshooting for gel-related problems.

Problem	Possible Reason	Solution
The gel is either too soft or does not polymerize.	Inhibition of polymerization reaction by oxygen.	Mix gel solution and gel components by gentle inversion or pipetting only. Work quickly while the gel solution on the microscope slide is exposed (i.e., without the top coverslip).
	Regents have degraded in quality.	Refrain from refreezing thawed aliquots of 10% APS. Once thawed, store aliquots at 4 °C for up to 1 week. Alternatively, make 10% APS fresh. Check all reagents, especially TEMED and APS, to ensure that they have not degraded in quality.
	The number of initiators added is too low.	Titrate up the amount of TEMED and 10% APS added to the gel solution in increments of 1 μL per 1 mL of gel solution.
Gel polymerizes too fast.	The number of initiators or the gel percentage is too high.	Titrate down the amount of 40% acrylamide solution added to the final gel solution in increments of 25 μL per 1 mL of gel solution (1% acrylamide decrease). Titrate down the amount of TEMED and 10% APS added to the gel solution in increments of 1 μL per 1 mL of gel solution.
Fluorescence is only detected on the surface of the brain.	The gel is too thick.	Reduce the stack height of supporting coverslips from 2 to 1. If this is not possible, attempt to reduce the gel concentration by titrating down the amount of 40% acrylamide solution added to the final gel solution in increments of 25 μL per 1 mL of gel solution (1% acrylamide decrease).

**Table 2:** Reagents used in the protocol.

Reagents	Components	Details
Acrylamide solution, 40%	40% acrylamide/bis-acrylamide, 19:1	For 1 mL: 400 mg acrylamide/bis-acrylamide, 19:1 Top up with DEPC H_2_O Store at 4 °C, away from light.
Ammonium persulfate, 10%(10% APS)	10% ammonium persulfate	For 1 mL: 0.1 g ammonium persulfate Top up with DEPC H_2_O Aliquots can be stored at −20 °C. After thawing, store at 4 °C for up to 1 week.
Amplification buffer	5× SSCT 5% dextran sulfate	For 40 mL: 10 mL 20× SSC 40 μL TWEEN 20 4 mL 50% dextran sulfate Top up with DEPC H_2_O
DAPI buffer	5 µg/mL DAPI 5× SSCT	For 1 mL: 5 μL 1 mg/mL DAPI in DMSO Top up with 5× SSCT Prepare fresh before use.
DAPI in DMSO, 1 mg/mL	DMSO 1 mg/mL DAPI	For 1 mL: 1 mg of DAPI Top up with DMSO Store at 4 °C, away from light.
DEPC H_2_O	0.1% DEPC	For 1 L: 1 mL DEPC Top up with MilliQ water Mix well and store in the dark overnight. Autoclave on the next day.
Detergent solution	150.0 mM NaCl 50.0 mM Tris-HCl, pH 7.5 2.5% TWEEN 20 1.0 mM EDTA, pH 8.0 1.0% sodium dodecyl sulfate	For 50 mL: 1.50 mL 5.0 M NaCl 2.50 mL 1.0 M Tris-HCl, pH 7.5 1.25 mL TWEEN 20 0.10 mL 0.5 M EDTA, pH 8.0 5.00 mL 10% sodium dodecyl sulfate Top up with DEPC H_2_O
Dextran sulfate, 50%	50% dextran sulfate	For 40 mL: 20 g dextran sulfate Top up with DEPC H_2_O
EDTA, 0.5 M, pH 8.0	0.5 M EDTA	For 500 mL: 73.06 g EDTA 300 mL MilliQ water Slowly adjust the pH to 8.0 with NaOH pellets. Top up with MilliQ water. Autoclave.
Gel solution (8% polyacrylamide)	8% acrylamide/bis-acrylamide, 19:1 800.0 mM NaCl 60.0 mM Tris-HCl, pH 7.5 0.4% TEMED (N,N,N′,N′ -Tetramethylethylenediamine) 0.1% APS	For 1 mL: 200 μL 40% acrylamide solution 400 μL 2.0 M NaCl 60 μL 1.0 Tris-HCl, pH 7.5 325 μL DEPC H_2_O 4 μL TEMED 10 μL 10% APS TEMED and APS should be added last, when the user is ready for the gel to polymerize. Prepare fresh before use.
Glycerol, 60%	60% glycerol	For 10 mL:∙ 6 mL glycerol∙ Top up with DEPC H_2_O
Phosphate-buffered formaldehyde, 4% (4% PFA)	1× PBS 4% formaldehyde 0.1% TWEEN 20	For 500 mL: 55 μL 37% formaldehyde Top up with 1× PBST Prepare fresh before use.
Phosphate-buffered saline, 1×(1× PBS)	140.0 mM NaCl 3.9 mM KH_2_PO_4_ 6.1 mM K_2_HPO_4_	For 50 mL: 5 mL 10× PBS Top up with DEPC H_2_O
Phosphate-buffered saline, 10×(10× PBS)	1.4 M NaCl 38.8 mM KH_2_PO_4_ 61.3 mM K_2_HPO_4_	For 1 L: 81.8 g NaCl 5.28 g KH_2_PO_4_ 10.68 g K_2_HPO_4_ Top up with MilliQ H_2_O and autoclave. Store at RT for up to 6 months.
Phosphate-buffered saline with TWEEN 20, 1×(1× PBST)	1× PBS 0.1% TWEEN 20	For 50 mL: 5 mL 10× PBS 50 μL TWEEN 20 Top up with DEPC H_2_O
Primary oligo mixture	30% probe hybridization buffer 0.8 μM of each primary oligo set	For each primary oligo set, up to 10 pairs of oligonucleotides, each at 100 μM, were pooled into a single master mix using 100 μL of each oligo. For 1 mL: 8 μL of each primary oligo set Top up with 30% probe hybridization buffer Prepare fresh before use.
Probe hybridization buffer, 30%	5× SSC 30% formamide 9 mM citric acid, pH 6.0 0.1% TWEEN 20 50 µg/mL heparin 1× Denhardt’s solution 5% dextran sulfate	For 40 mL: 10 mL 20× SSC 12 mL formamide 360 μL 1 M citric acid, pH 6.0 40 μL TWEEN 20 200 μL 10 mg/mL heparin 800 μL 50× Denhardt’s solution 4 mL 50% dextran sulfate Top up with DEPC H_2_O Store at 4 °C for up to 1 month.
Probe wash buffer, 30%	5× SSC 30% formamide 9 mM citric acid, pH 6.0 0.1% TWEEN 20 50 µg/mL heparin	For 40 mL: 10 mL 20× SSC 12 mL formamide 360 μL 1 M citric acid, pH 6.0 40 μL TWEEN 20 200 μL 10 mg/mL heparin Top up with DEPC H_2_O Store at 4 °C for up to 1 month.
Saline-sodium citrate buffer, 5× (5× SSC)	150.0 mM NaCl 17.1 mM trisodium citrate	For 40 mL: 10 mL 20× SSC Top up with DEPC H_2_O
Saline-sodium citrate buffer, 20×(20× SSC)	3.0 M NaCl 341.8 mM trisodium citrate	For 1 L: 175.3 g NaCl 88.2 g trisodium citrate Top up with MilliQ H_2_O and autoclave. Store at RT for up to 6 months.
Saline-sodium citrate buffer with TWEEN 20, 5×(5× SSCT)	5× SSC 0.1% TWEEN 20	For 40 mL: 10 mL 20× SSC 40 μL TWEEN 20 Top up with DEPC H_2_O
SDS, 10%	10% SDS	For 50 mL: 5 g SDS Top up with DEPC H_2_O
Secondary oligo mixture	Amplification buffer 1.7% of each H1 hairpins 1.7% of each H2 hairpins	Snap-cool each stock H1 and H2 hairpins separately by heating to 95 °C for 90 s, then allow to cool to RT in a dark environment for 30 min. For 600 μL: 10 μL of each hairpin, snap-cooled Top up with amplification buffer Prepare fresh before use.
Signal removal solution	0.1 M Tris-HCl, pH 7.5 25.0 mM MgCl_2_ 2.5 mM CaCl_2_ 15 mU/μL DNase I	For 1 mL: 100 μL 1.0 M Tris-HCl, pH 7.5 50 μL 0.5 M MgCl_2_ 5 μL 0.5 M CaCl_2_ 15 μL 1 U/μL DNaseI Top up with DEPC H_2_O Prepare fresh before use.
Signal wash buffer 1	0.1 M Tris-HCl, pH 7.5 25.0 mM MgCl_2_ 2.5 mM CaCl_2_	For 10 mL: 1 mL 1.0 M Tris-HCl, pH 7.5 500 μL 0.5 M MgCl_2_ 50 μL 0.5 M CaCl_2_ Top up with DEPC H_2_O
Signal wash buffer 2	0.1 M Tris-HCl, pH 7.5 25.0 mM MgCl_2_ 2.5 mM CaCl_2_ 2.5 mM EDTA, pH 8.0 1.5% sodium dodecyl sulfate	For 1 mL: 100 μL 1.0 M Tris-HCl, pH 7.5 50 μL 0.5 M MgCl_2_ 5 μL 0.5 M CaCl_2_ 5 μL 0.5 M EDTA, pH 8.0 150 μL 10% sodium dodecyl sulfate Top up with DEPC H_2_O

## Data Availability

All additional data mentioned in the protocol are mentioned in the Supplementary Materials.

## Supplementary Materials

The following supporting information can be downloaded at: https://www.mdpi.com/article/10.3390/mps8020031/s1, Figure S1: Nuclear staining (DAPI) across a 40 µm section of a *B. anynana* larval brain. Dotted lines indicate the connective tissue.; Figure S2 Expression of omb, Dll and synapsin in a larval brain of Bicyclus anynana at different optical sections and showing single-cell resolution. Sections shown are obtained at z heights of (A) 0 µm, (B) 5 µm, (C) 10 µm, (D) 15 µm, (E) 20 µm, (F) 25 µm, (G) 30 µm, with highlighted section (G’) zoomed in, (H) 35 µm, with highlighted section (H’) zoomed in, and (I) 40 µm, with highlighted section (I’) zoomed in; Figure S3: Expression of optomoter blind (omb), Distal-less (Dll), and synapsin in a larval brain of *B. anynana*. Dotted lines indicate con-nective tissues. (A-E) Anterior domain of the larval brain, showing signals for (A) omb, (B) Dll, (C) Synapsin, (D) DAPI, and (E) merged channels, with highlighted sections zoomed in. (F–I) Posterior domain of the larval brain, showing signals for (F) omb, (G) Dll, (H) Synapsin, (I) DAPI, and (J) merged channels, with highlighted sections zoomed in; Figure S4: Expression of optomotor blind (omb) and Synapsin across a 75 µm section of a *B. anynana* larval brain left lobe; Figure S5: Expression of zinc finger protein SNAI2-like (SNAI2), extradenticle (exd) and acetyl coenzyme A acetyltransferase B (AcatB) across a 95 µm section of a *B. anynana* larval brain left lobe; Figure S6: Expression of dachshund (dac) and yellow-like across a 110 µm section of a *B. anynana* larval brain right lobe; Figure S7: Expression of wry and bric-a-bac 1 (bab1) across a 75 µm section of a *B. anynana* larval brain left lobe; Figure S8: Expression of omb, Synapsin, and Dll across 40 µm section of a *B. anynana* larval brain (posterior domain); Video S1: Three-dimensional expression of omb (red), Synapsin (green), Dll (gray), and DAPI (blue) in the larval brain (anterior domain) of *B. anynana*; Video S2: Three-dimensional expression of omb (red), Synapsin (green), Dll (gray), and DAPI (blue) in larval brain (posterior domain) of *B. anynana*; Video S3: Three-dimensional expression of omb in the larval brain (left lobe) of B. any-nana (DAPI in blue); Video S4: Three-dimensional expression of Synapsin in the larval brain (left lobe) of *B. anynana* (DAPI in blue); Video S5: Three-dimensional expression of Dll in the larval brain (left lobe) of B. any-nana (DAPI in blue); Video S6: Three-dimensional expression of SNAI2 in the larval brain (left lobe) of *B. anynana* (DAPI in blue); Video S7: Three-dimensional expression of exd in the larval brain (left lobe) of B. any-nana (DAPI in blue); Video S8: Three-dimensional expression of AcatB in the larval brain (left lobe) of *B. anynana* (DAPI in blue); Video S9: Three-dimensional expression of dac in the larval brain (left lobe) of B. any-nana (DAPI in blue); Video S10: Three-dimensional expression of yellow-like in the larval brain (left lobe) of *B. anynana* (DAPI in blue); Video S11: Three-dimensional expression of wry in the larval brain (left lobe) of *B. anynana* (DAPI in blue); Video S12: Three-dimensional expression of bab1 in the larval brain (left lobe) of *B. anynana* (DAPI in blue).
