# Supplementary material for: Mapping Gene Expression in Whole Larval Brains of Bicyclus anynana Butterflies
Source: Methods Protoc. 2025 Mar 13;8(2):31. doi: 10.3390/mps8020031 (PMC11932290; doi:10.3390/mps8020031)
Supplement: Supplementary file 1 [file mps-08-00031-s001.zip › (supp1)_Larval_Brain_Bicyclus_v15.0.pdf]

Protocol

Mapping Gene Expression in Whole Larval Brains of *Bicyclus anynana* Butterflies

Tirtha Das Banerjee *,†, Linwan Zhang † and Antónia Monteiro *

Department of Biological Sciences, National University of Singapore, 14 Science Drive 4, Singapore 117543, Singapore; zhang.linwan@u.nus.edu
* Correspondence: tirtha_banerjee@u.nus.edu (T.D.B.); antonia.monteiro@nus.edu.sg (A.M.)
† These authors contributed equally to this work.

Supplementary file 1

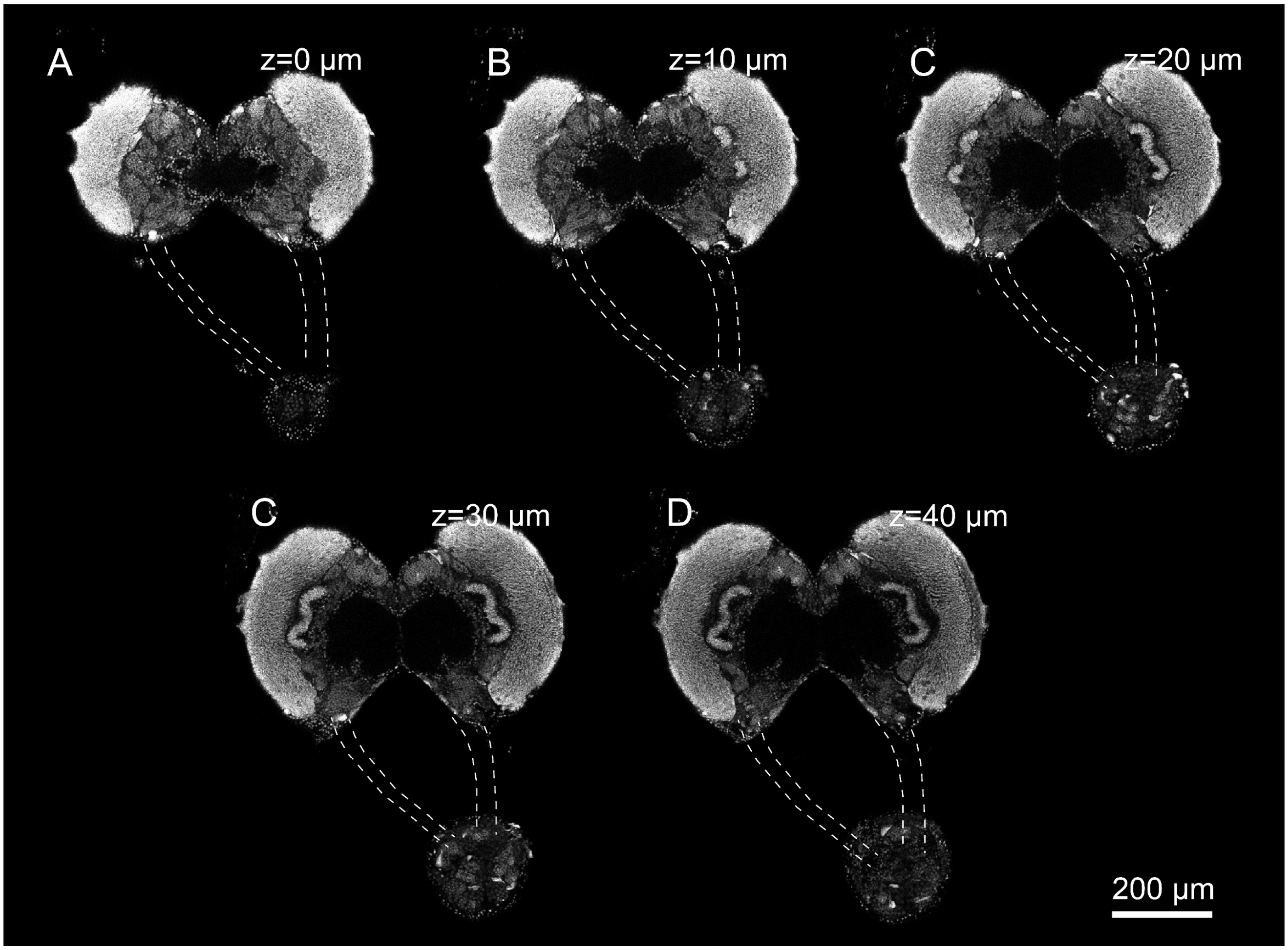

Figure S1. Nuclear staining (DAPI) across a 40 μm section of a *B. anynana* larval brain. Dotted lines indicate the connective tissue.

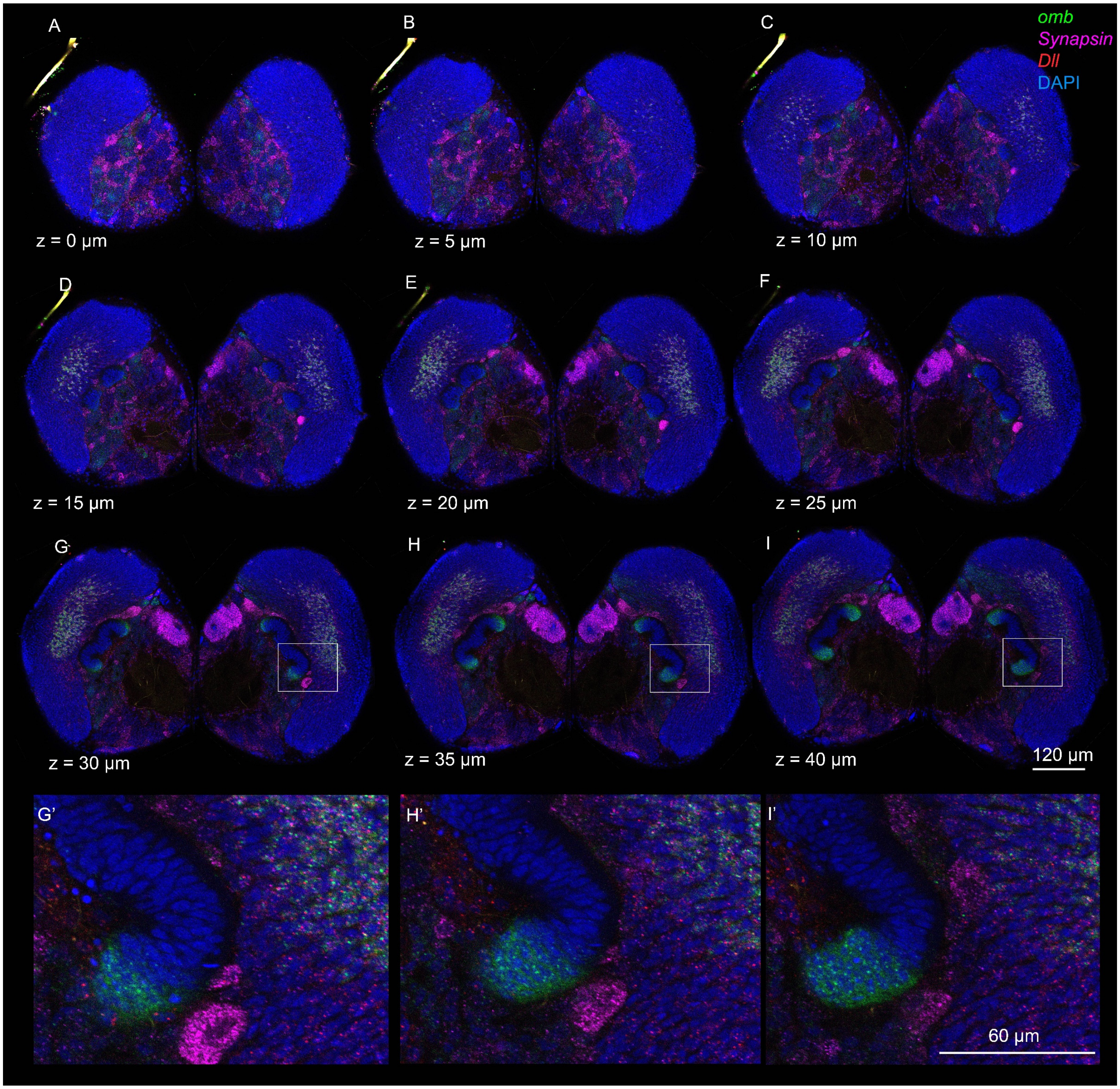

Figure S2. Expression of *omb, Dll* and *synapsin* in a larval brain of *Bicyclus anynana* at different optical sections and showing single-cell resolution. Sections shown are obtained at z heights of (**A**) 0 µm, (**B**) 5 µm, (**C**) 10 µm, (**D**) 15 µm, (**E**) 20 µm, (**F**) 25 µm, (**G**) 30 µm, with highlighted section (**G′**) zoomed in, (**H**) 35 µm, with highlighted section (**H′**) zoomed in, and (**I**) 40 µm, with highlighted section (**I′**) zoomed in.

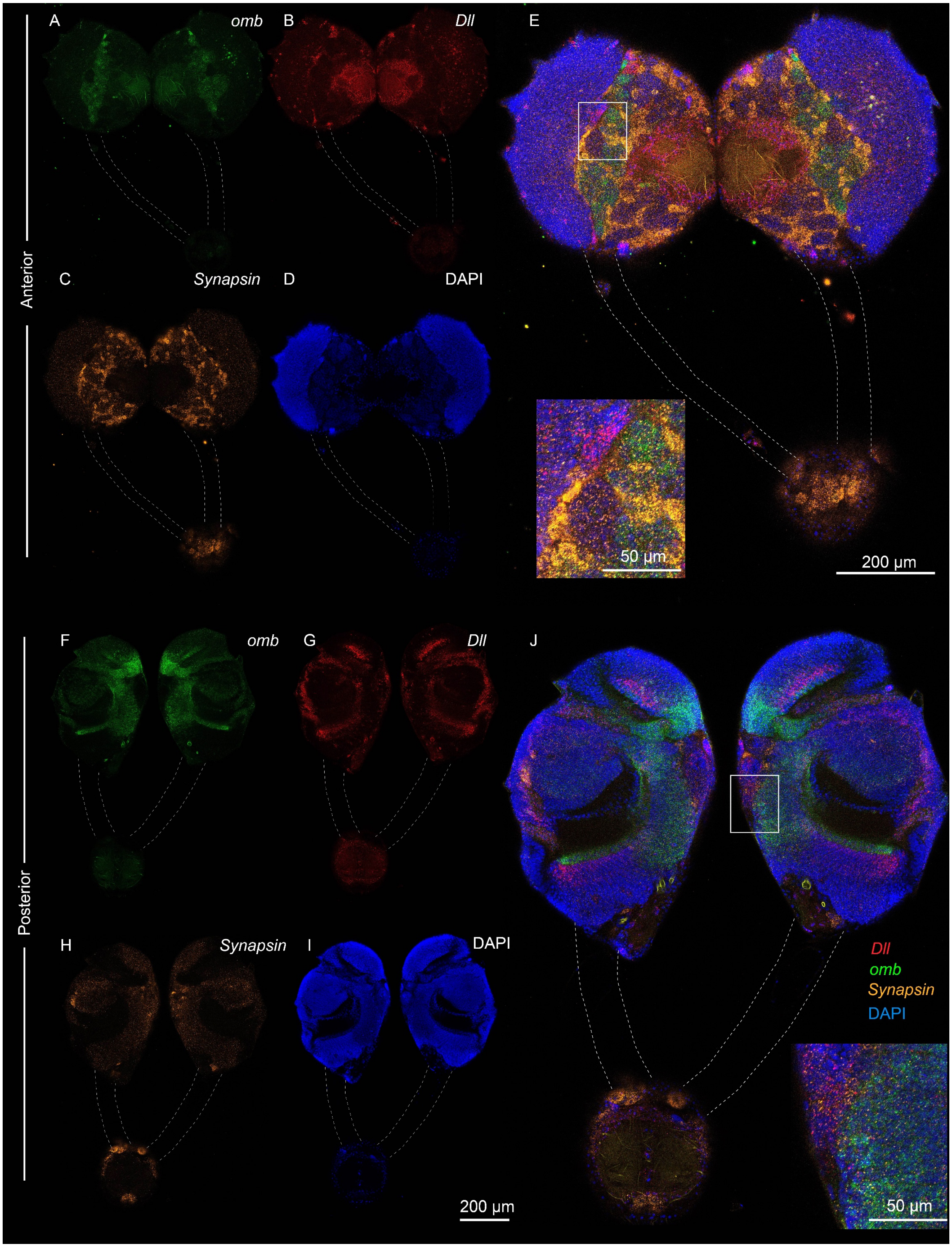

Figure S3. Expression of *optomoter blind* (*omb*), *Distal-less* (*Dll*), and *synapsin* in a larval brain of *B. anynana*. Dotted lines indicate connective tissues. (**A-E**) Anterior domain of the larval brain, showing signals for (**A**) *omb*, (**B**) *Dll*, (**C**) *Synapsin*, (**D**) DAPI, and (**E**)

merged channels, with highlighted sections zoomed in. (**F-I**) Posterior domain of the larval brain, showing signals for (**F**) *omb*, (**G**) *Dll*, (**H**) *Synapsin*, (**I**) DAPI, and (**J**) merged channels, with highlighted sections zoomed in.

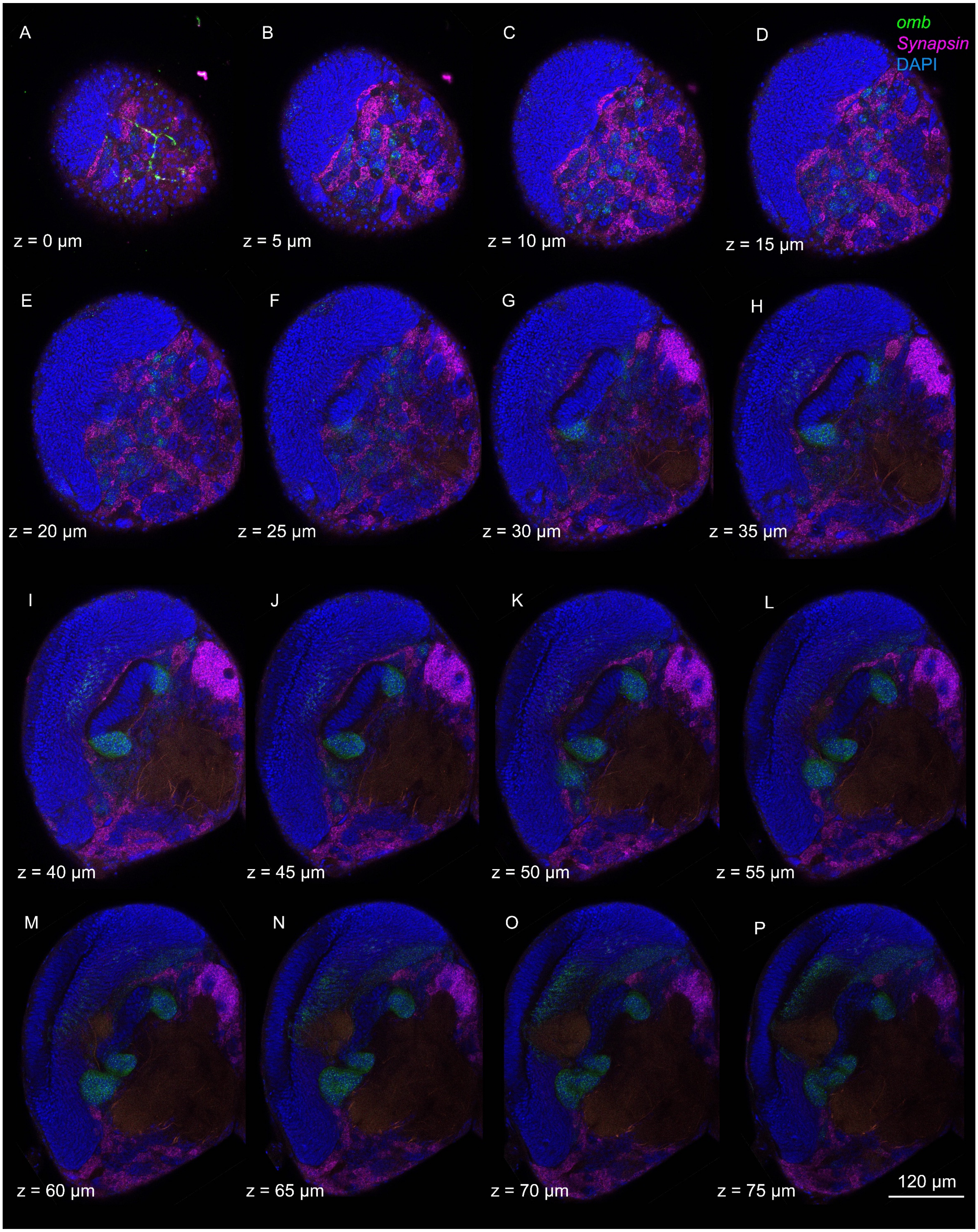

Figure S4. Expression of *optomotor blind* (*omb)* and *Synapsin* across a 75 µm section of a *B. anynana* larval brain left lobe.

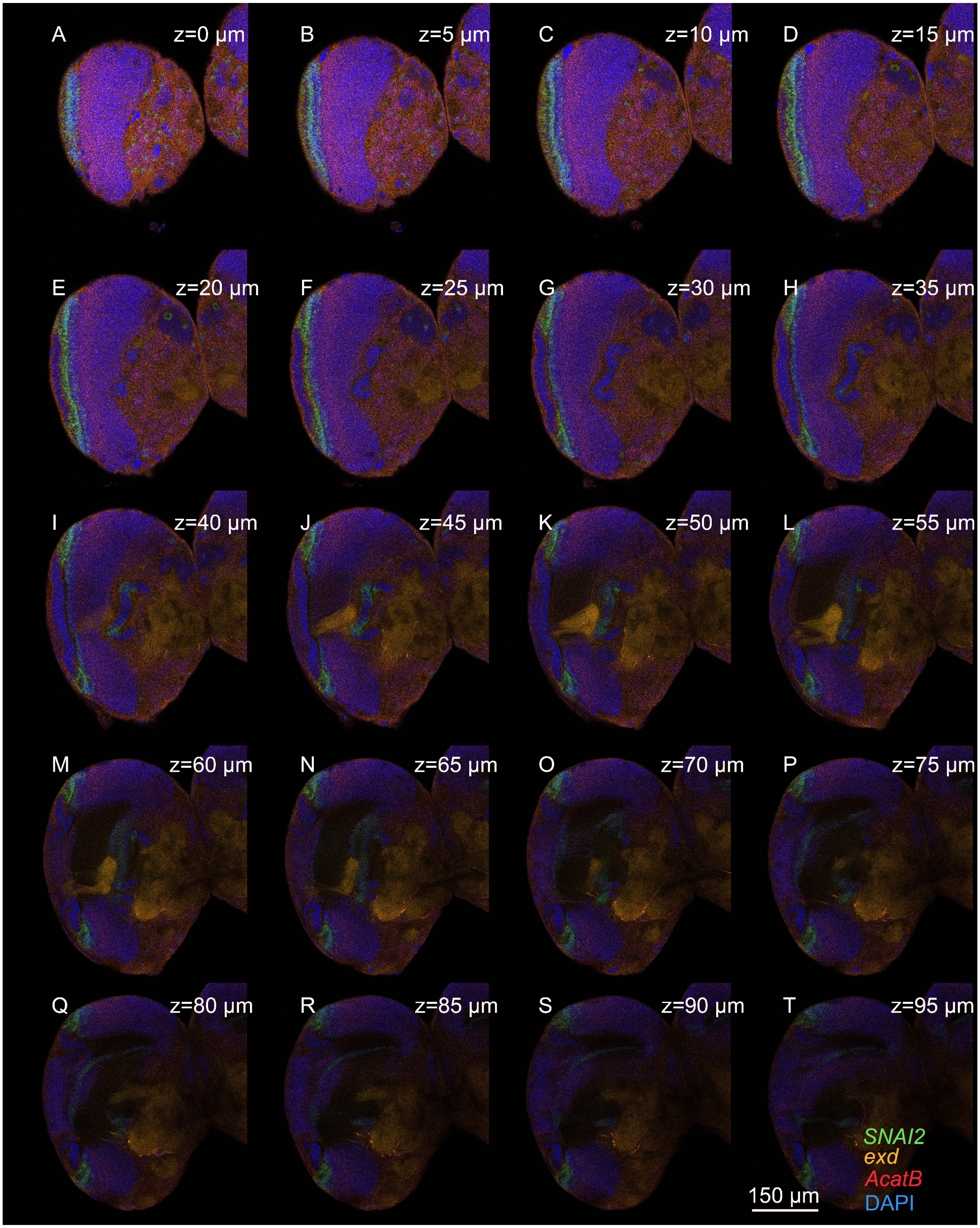

Figure S5. Expression of *zinc finger protein SNAI2-like (SNAI2), extradenticle (exd)* and *acetyl coenzyme A acetyltransferase B (AcatB)* across a 95 µm section of a *B. anynana* larval brain left lobe.

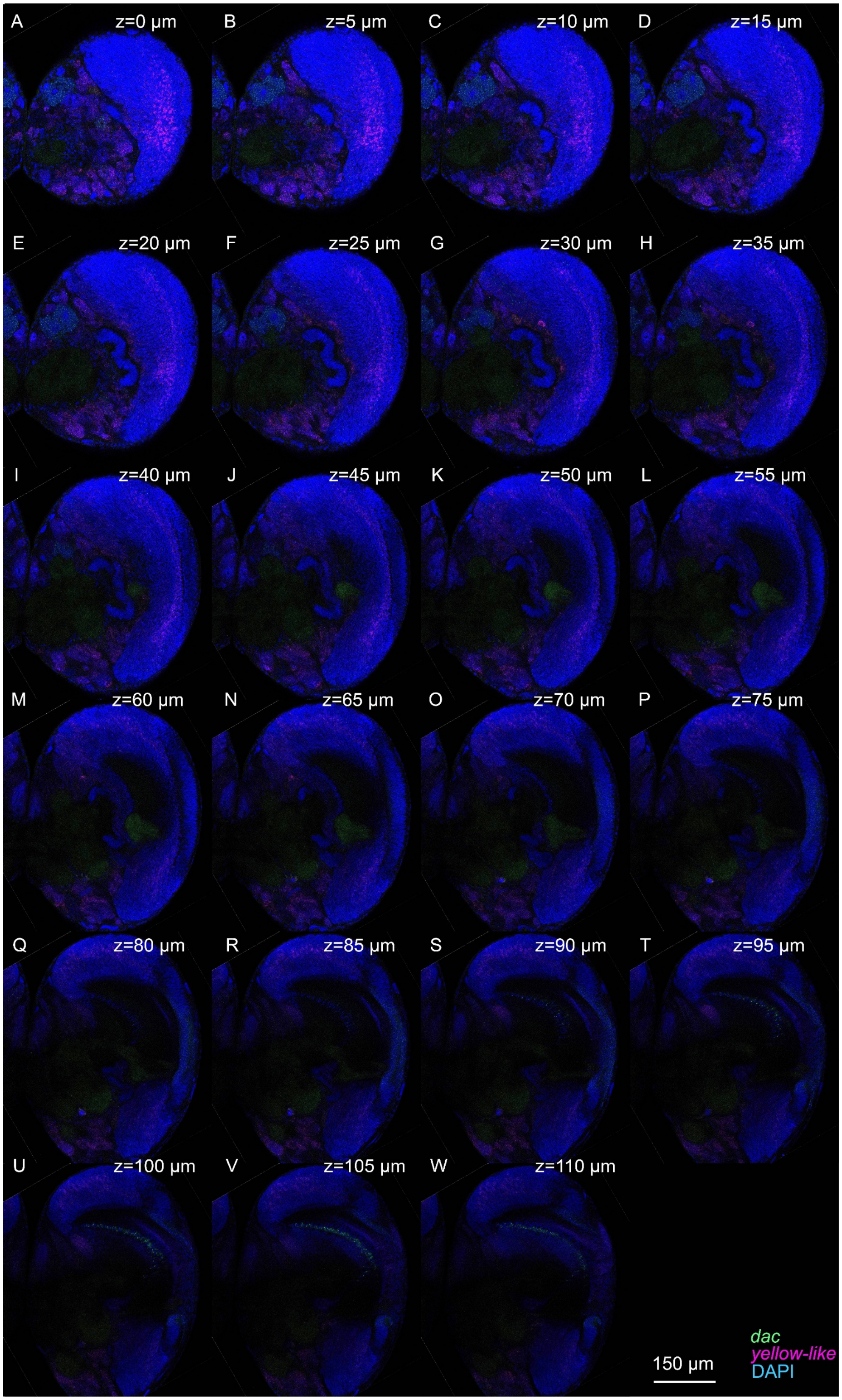

Figure S6. Expression of *dachshund* (*dac)* and *yellow-like* across a 110 µm section of a *B. anynana* larval brain right lobe.

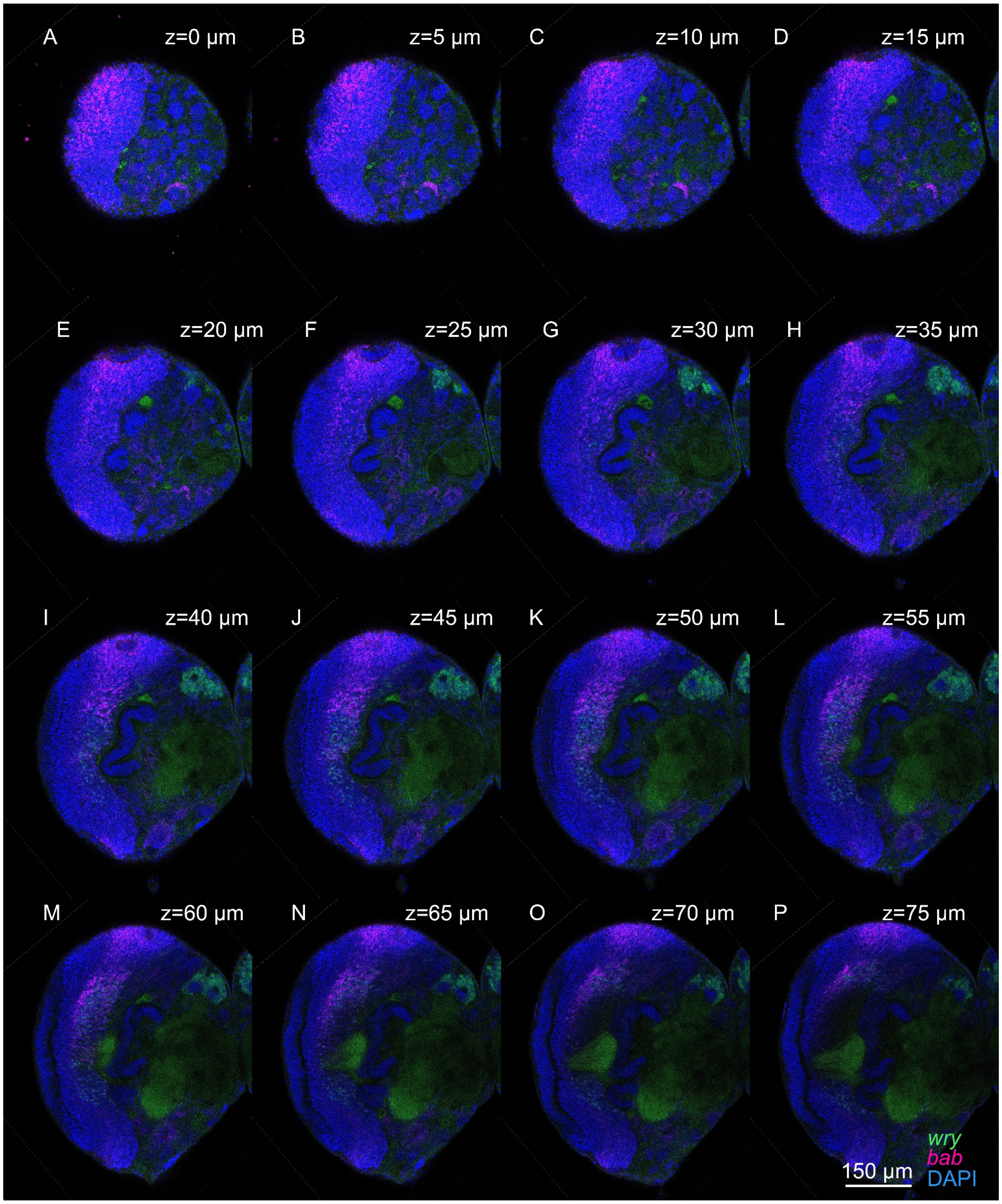

Figure S7. Expression of *wry* and *bric-a-bac 1* (*bab1)* across a 75 µm section of a *B. anynana* larval brain left lobe.

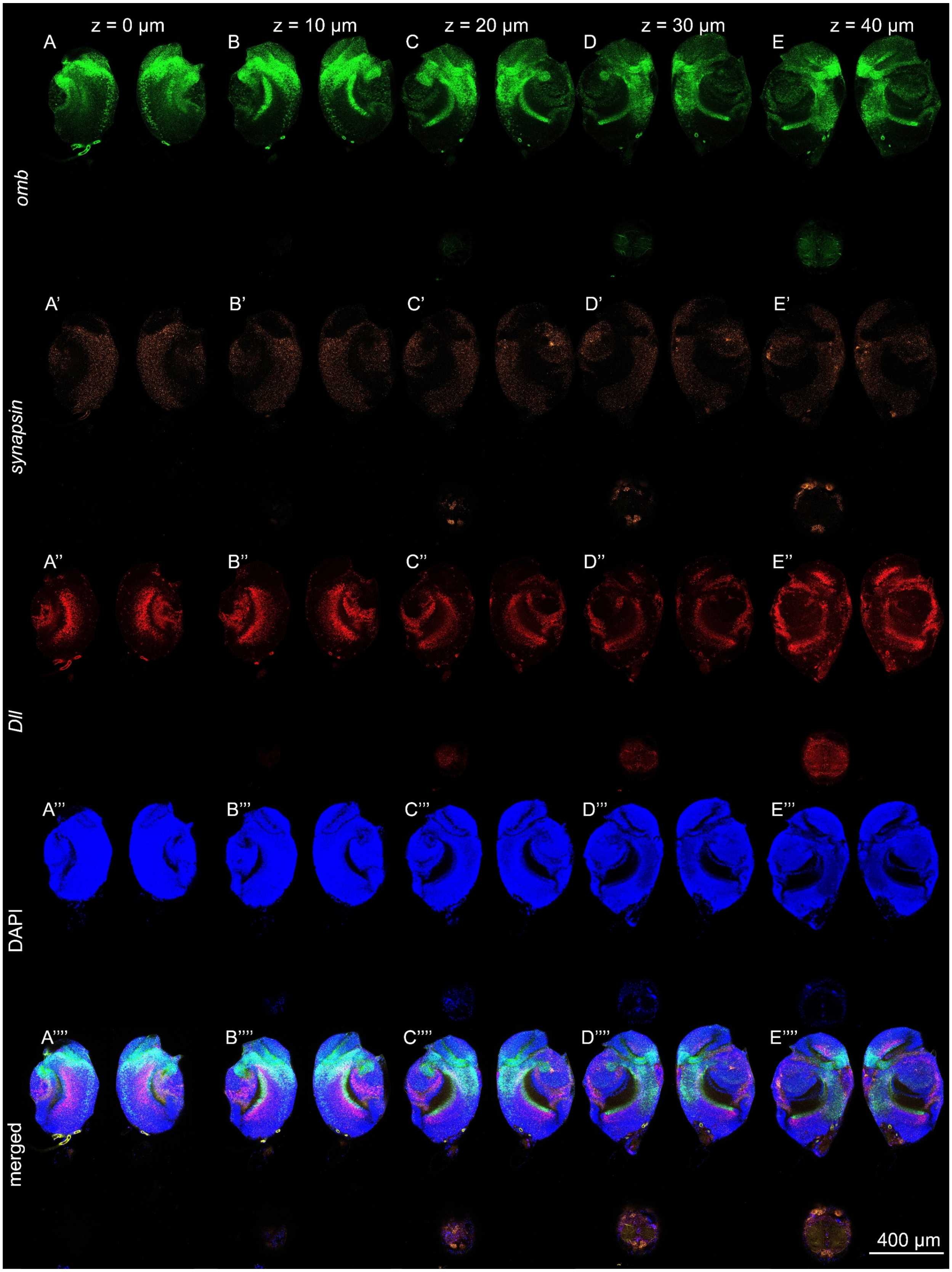

Figure S8. Expression of *omb, Synapsin,* and *Dll* across 40 µm section of a *B. anynana* larval brain (posterior domain).
